# Microrna-221 and Microrna-222 Modulate Differentiation and Maturation of Skeletal Muscle Cells

**DOI:** 10.1371/journal.pone.0007607

**Published:** 2009-10-27

**Authors:** Beatrice Cardinali, Loriana Castellani, Pasquale Fasanaro, Annalisa Basso, Stefano Alemà, Fabio Martelli, Germana Falcone

**Affiliations:** 1 Istituto di Biologia Cellulare, Consiglio Nazionale delle Ricerche, Monterotondo, Italy; 2 Dipartimento di Scienze Motorie e della Salute, Università degli Studi di Cassino, Cassino, Italy; 3 IRCCS- Policlinico San Donato, San Donato Milanese, Milan, Italy; 4 Laboratorio di Patologia Vascolare, Istituto Dermopatico dell'Immacolata, IRCCS, Rome, Italy; The University of Hong Kong, Hong Kong

## Abstract

**Background:**

MicroRNAs (miRNAs) are a class of small non-coding RNAs that have recently emerged as important regulators of gene expression. They negatively regulate gene expression post-transcriptionally by translational repression and target mRNA degradation. miRNAs have been shown to play crucial roles in muscle development and in regulation of muscle cell proliferation and differentiation.

**Methodology/Principal Findings:**

By comparing miRNA expression profiling of proliferating myoblasts versus differentiated myotubes, a number of modulated miRNAs, not previously implicated in regulation of myogenic differentiation, were identified. Among these, miR-221 and miR-222 were strongly down-regulated upon differentiation of both primary and established myogenic cells. Conversely, miR-221 and miR-222 expression was restored in post-mitotic, terminally differentiated myotubes subjected to Src tyrosine kinase activation. By the use of specific inhibitors we provide evidence that expression of miR-221 and miR-222 is under the control of the Ras-MAPK pathway. Both in myoblasts and in myotubes, levels of the cell cycle inhibitor p27 inversely correlated with miR-221 and miR-222 expression, and indeed we show that p27 mRNA is a direct target of these miRNAs in myogenic cells. Ectopic expression of miR-221 and miR-222 in myoblasts undergoing differentiation induced a delay in withdrawal from the cell cycle and in myogenin expression, followed by inhibition of sarcomeric protein accumulation. When miR-221 and miR-222 were expressed in myotubes undergoing maturation, a profound alteration of myofibrillar organization was observed.

**Conclusions/Significance:**

miR-221 and miR-222 have been found to be modulated during myogenesis and to play a role both in the progression from myoblasts to myocytes and in the achievement of the fully differentiated phenotype. Identification of miRNAs modulating muscle gene expression is crucial for the understanding of the circuits controlling skeletal muscle differentiation and maintenance.

## Introduction

Skeletal myogenesis requires the occurrence of specific coordinated events, including exit from the cell cycle, transcription of muscle-specific proteins, fusion into polynucleated fibers and assembly of the contractile apparatus. Such complex processes are regulated at multiple levels. Determination and differentiation pathways are under the control of the MyoD family of myogenic regulatory factors (MRFs) that cooperate with members of the myocyte enhancer factor-2 family of transcription factors to synergistically activate muscle-specific gene transcription by recruiting chromatin remodeling proteins [1], [2]. A fundamental role in establishing and maintaining the post-mitotic state of differentiated cells is played by cyclin-dependent kinase inhibitors (CDKIs) such as p21, p27 and p57 that function by coupling cell cycle arrest and cell differentiation [3]. Moreover, there is evidence for the existence of a functional cross-talk between CDKIs and MRFs [4], [5], critical for induction of myogenesis.

Recent studies have identified the post-transcriptional control of gene expression as a crucial level of regulation of myogenesis. Among the critical mediators of such control, an important role is played by miRNAs, small non coding RNAs that specifically bind the 3′untranslated regions (3′UTRs) of mRNAs and control their stability and translational efficiency [6], [7]. Several miRNAs have been identified, some of which, miR-1, miR-133a and miR-206, are expressed specifically in muscle tissue [8], [9]. The binding of MRFs to the presumptive promoters of muscle-restricted miRNAs, together with the over-expression and knock-down of these miRNAs in muscle tissues and in myogenic cell lines [10], [11], have provided experimental support for their role in muscle differentiation. Interestingly, miR-1 and miR-206 promote myogenesis by targeting transcriptional repressors of muscle gene expression, whereas miR-133 inhibits myogenesis by enhancing myoblast proliferation [12], [13]. Little is known on how extracellular signals impinge on the regulation of miRNAs involved in myogenic differentiation.

Expression of oncogenes or exogenous growth factors has been shown to interfere with myogenic differentiation by modulating various extracellular-signal activated pathways involved in regulation of skeletal muscle differentiation [14]. Activation of the p38 mitogen activated protein kinase (MAPK) pathway promotes muscle differentiation, while its inhibition prevents expression of muscle-specific genes and fusion of myocytes [15]. Oncogenic activation of the Ras-MAPK pathway, instead, inhibits muscle differentiation in most cell models studied, whereas inhibition of endogenous MEK usually favors differentiation [16], [17]. Transformation of quail embryo myoblasts with temperature-sensitive mutants of the v-*src* oncogene (QMb-ts) allows cells to proliferate in low mitogen medium at the permissive temperature for the Src kinase and to fully differentiate into myotubes that assemble highly ordered sarcomeric structures at the restrictive temperature [18]. The block of differentiation of quail myoblasts transformed by ts-Src is mainly due to the constitutive activation of Ras-MAPK and inhibition of p38 MAPK pathways [16]. A unique property of this cell context is that the ts kinase can be reactivated in terminally differentiated myotubes leading to marked changes in muscle-specific mRNA stability and prominent defects in the assembly of contractile proteins [18], [19].

In this study, we made use of the QMb-ts myoblast model, which faithfully reproduces myogenesis *in vitro*, in order to identify novel miRNAs that are specifically modulated in both differentiation and maturation of skeletal muscle cells. Two of them, miR-221 and miR-222, are down-regulated upon differentiation of avian and mammalian myoblasts and are under the control of the Ras-MAPK signaling pathway. We show that p27 is a target of miR-221 and miR-222 in myoblasts and in myotubes subjected to Src activation and that alterations of their expression lead to defects in the transition from myoblasts to myocytes and in the assembly of sarcomeres in myotubes.

## Results

### microRNA expression profiling upon myogenic differentiation

**Figure 1:**
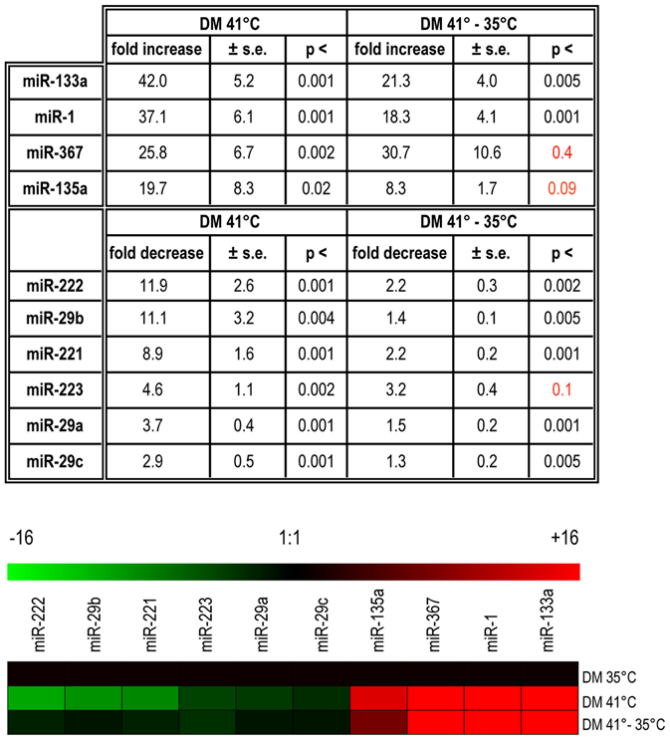
Profiling of modulated miRNAs in QMb-ts. miRNA expression was measured in QMb-ts, cultivated either at 35°C or 41°C for 48 hours and in QMb-ts maintained at 41°C for 30 hours and shifted to 35°C for additional 18 hours. Values in table were normalized according to median values and expressed as a function of values at 35°C DM using the comparative Ct method. Standard error (s.e.) and statistical significance (p, n = 6) are shown. P values in red are not statistically significant. Heat map representing miRNA modulation is expressed in a linear scale with a maximum value of 16 fold to highlight differences within this range. Green and red luts indicate down- and up-regulation, respectively.

Polyclonal populations of QMb-ts cells can be kept proliferating both in growing (GM) and differentiation medium (DM) at the permissive temperature for the v-Src kinase (35°C) and can synchronously differentiate into multinucleate myotubes exhibiting a high degree of fusion (over 90%) and structural maturation when shifted to the restrictive temperature (41°C). In addition, the ts kinase can be reactivated in myotubes by a simple temperature shift (from 41°C to 35°C) resulting in profound alterations of the differentiated phenotype [18]. To search for miRNAs specifically modulated during the progression from myoblasts to myotubes and in myotubes subjected to Src-induced perturbation, a miRNA expression profile was determined by quantitative real-time PCR in proliferating myoblasts maintained at 35°C in DM, in myoblasts allowed to differentiate for 48 hours at 41°C in DM, and myotubes differentiated at 41°C for 30 hours and then shifted to 35°C for 18 hours (Table S1). Novel miRNAs, not previously described as linked to myogenic differentiation, were identified and, among them, we chose all miRNAs showing a degree of modulation between myoblasts and myotubes bigger than eight-fold, and a selected group of microRNAs modulated to a lesser extent (Fig. 1). Expression of miR-16, previously found to be unaffected by muscle differentiation, was used to normalize different samples and microRNA modulation was expressed as fold increase or decrease in myotubes, compared to myoblasts. Two miRNAs, miR-135a and miR-367, were strongly up-regulated in differentiated myotubes to an extent comparable to that measured for miR-1 and miR-133a, previously shown to be specifically up-regulated in differentiated murine muscle cells [12]. Upon Src activation in myotubes, these miRNAs, with the exception of miR-367, were moderately down-regulated. On the contrary, miR-221, miR-222 and miR-29b were down-modulated in differentiated myotubes. Other members of the miR-29-family, namely miR29a and miR29c, although occupying chromosomal positions adjacent to miR-29b, were down-modulated to a lesser extent. Consistent with our results, a slight down-modulation of miR-29 family members has been previously reported upon differentiation of C2C12 mouse myoblasts [12]. In contrast, these miRNAs have been found up-modulated upon differentiation of C2C12 and human myoblasts by another group [20], possibly due to different culture conditions. miR-223 was also included among the modulated miRNAs shown in Fig. 1 due to its previously recognized role in granulocytic differentiation [21]. Expression of miR-221, miR-222 and miR-29b, strongly reduced in myotubes, was induced upon shift to 35° to levels comparable to myoblasts, highlighting a possible role of these miRNAs in both myoblasts and myotubes subjected to Src-induced alteration.

**Figure 2:**
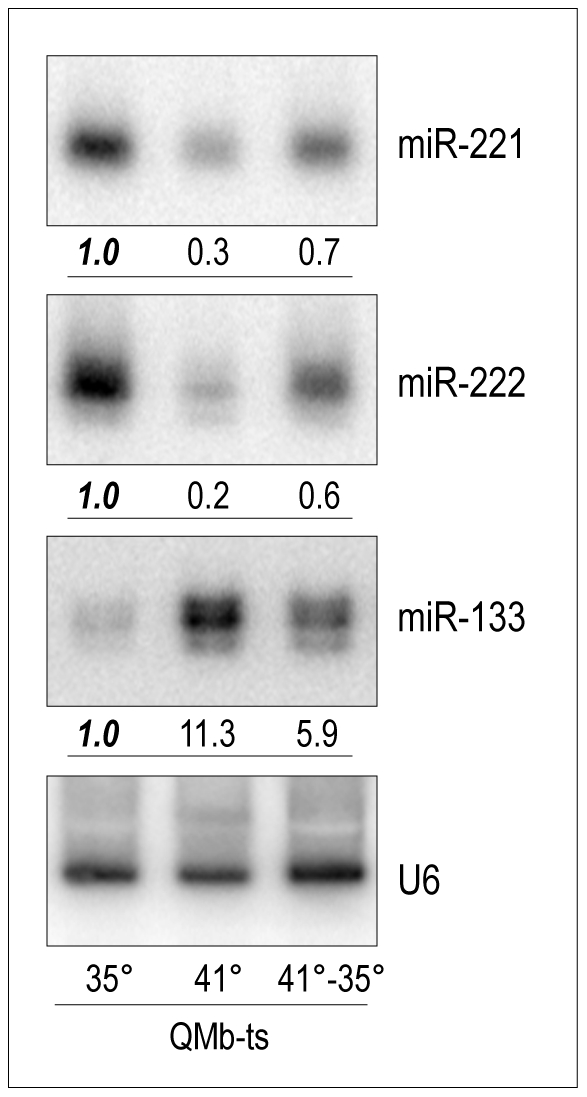
Expression of miR-221, miR-222 and muscle specific miR-133 in QMb-ts myoblasts. Northern blot analysis showing expression of miR-221 and miR-222, compared to muscle-specific miR-133, in QMb-ts myoblasts at 35°C, myotubes at 41°C and myotubes shifted back to 35°C. Numbers represent the quantity of each miR expressed as fold decrease/increase relative to that measured in proliferating myoblasts (35°), taken as 1.0. U6 snRNA was used for normalization.

Among the modulated miRNAs we pursued the study of miR-221 and miR-222 (miR-221/222), previously implicated in tumor cell proliferation [22]–[24], since little is known about their possible involvement in myogenesis. Northern blot analysis of miR-221/222 expression in QMb-ts cells confirmed that these miRNAs are highly expressed in proliferating myoblasts, down-regulated in differentiated myotubes and that their regulation is opposite to that of miR-133 (Fig. 2). In addition, their re-induction in quail myotubes following Src activation shows that they can be modulated independently of proliferation.

In order to establish their possible role in myogenic differentiation, modulation of miR-221/222 was analyzed in other myogenic cell contexts, such as primary quail myoblasts (QMb), primary mouse satellite cells (MSC) and two well characterized rodent cell lines: mouse C2C12 and rat L6C5 myoblasts. RNAs extracted from these cell types both in GM and DM at different time points were analyzed for the expression of miR-221/222 by quantitative PCR. As shown in Table 1, miR-221/222 were down-modulated upon terminal differentiation in all myogenic cells analyzed, whether primary myoblasts or established cell lines, albeit to different extents and with different kinetics, indeed suggesting their involvement in regulation of differentiation. While the modulation of miR-221/222 in avian myoblasts was pronounced, an average two-fold modulation was observed in all mammalian myoblasts analyzed, in keeping with previous reports in other cell contexts [25] where a similar modulation was shown to be functionally relevant. miR-1 was also analyzed as control.

**Table 1:** Modulation of miR-221, miR-222 and miR-1 expression upon myogenic differentiation.

	miR-221			miR-222			miR-1		
	fold decrease	±s.e.	p	fold decrease	±s.e.	p	fold increase	±s.e.	p
**QMb-ts**	8.0	1.2	0.001	10.1	1.7	0.001	37.1	6.1	0.001
**QMb**	4.8	1.5	0.001	6.2	1.9	0.001	52.0	18.7	0.01
**C2C12 2d**	2.0	1.4	0.04	2.5	0.7	0.001	245.8	108.2	0.04
**C2C12 4d**	1.5	0.4	0.06	2.0	0.5	0.001	179.7	64.1	0.05
**L6C5 2d**	1.5	0.2	0.009	1.5	0.3	0.009	30.3	28.1	0.13
**L6C5 4d**	2.0	0.5	0.001	2.2	0.3	0.001	242.2	46.3	0.004
**MSC 1d**	1.9	0.2	0.001	1.9	0.1	0.001	9.2	5.4	0.10
**MSC 2d**	1.1	0.3	0.29	1.6	0.4	0.04	25.9	9.4	0.02

RNAs extracted from QMb-ts and QMb induced to differentiate for 2 days, C2C12 mouse myoblasts and L6C5 rat myoblasts induced to differentiate for 2 (2d) and 4 (4d) days, and MSC induced to differentiate for 1 (1d) and 2 (2d) days were analyzed for the expression of miR-221, miR-222 and miR-1 by real-time RT-PCR. Fold decrease of miR-221 and miR-222 and fold increase of miR-1 expression in myotubes is shown for each cell type relative to levels detected in proliferating myoblasts. Standard error (s.e.) and statistical significance (p, n≥3; for QMb and MSC, n = 3; for L6C5, n = 4; for QMb-ts, n = 6; for C2C12 n = 6) are shown. Not significant p values are underlined.

### Expression of miR-221/222 inversely correlates with p27 accumulation and is under control of the Ras-MAPK pathway

**Figure 3:**
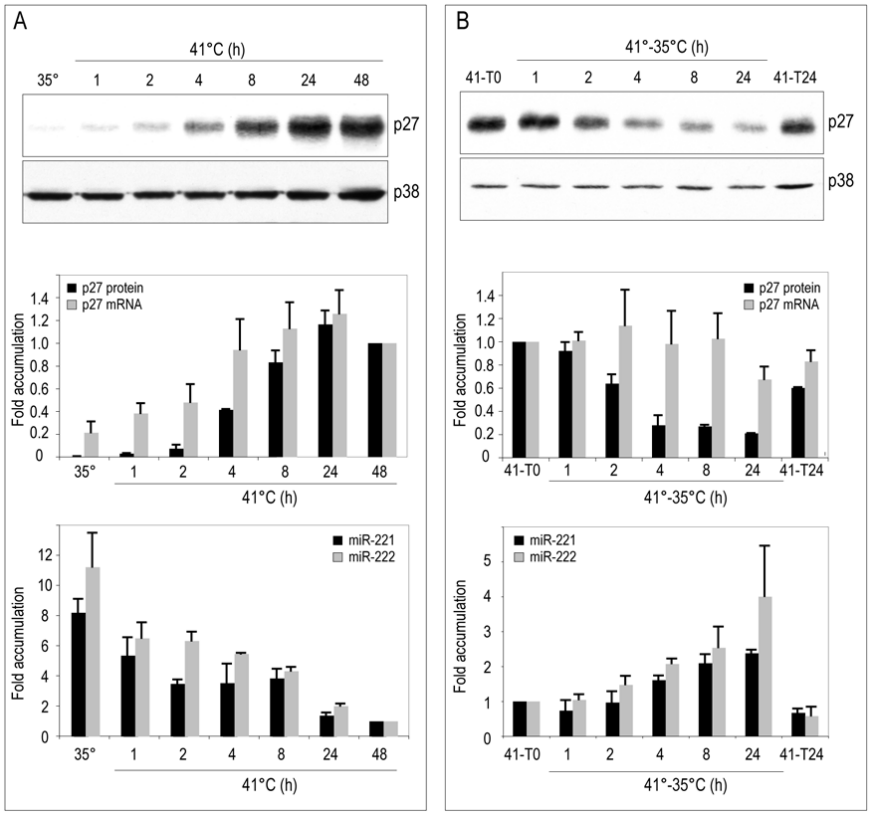
Kinetics of p27, miR-221 and miR-222 accumulation in QMb-ts upon induction of differentiation at 41°C and shift down of myotubes to 35°C. (A) QMb-ts myoblasts allowed to differentiate in DM upon temperature shift from 35°C to 41°C and (B) myotubes differentiated in DM for 40 hours at 41°C (41-T0) subjected to Src activation at 35°C were analysed at different time points for expression of p27 protein by Western blot (upper panels) and of p27 mRNA, miR-221 and miR-222 by real time RT-PCR. In B, 41-T24 represents control myotubes kept at 41°C for additional 24 hours. Accumulation levels of p27 protein and mRNA, miR-221 and miR-222, expressed relative to levels detected at 41°C, taken as 1.0, are shown in the histograms, normalized for p38 protein, GAPDH mRNA and miR-16 respectively. Error bars represent standard error (n = 3).

Being p27 CDKI a direct target of miR-221 and miR-222 in human cancer cells [22]–[24], we investigated whether there was a correlation between p27 and miR-221 and miR-222 expression in myogenic cells. Therefore, QMb-ts myoblasts induced to differentiate at 41°C or myotubes subjected to Src activation were analyzed at different time points for the expression of p27 protein and mRNA, and for the expression of miR-221/222. Upon shift to differentiation-permissive conditions, both p27 mRNA and protein expression were induced, whereas the expression of miR-221/222 was correspondingly reduced (Fig. 3A). In contrast, when Src was activated in myotubes, p27 protein levels were reduced up to 5 fold while p27 mRNA levels were barely affected (Fig. 3B), suggesting that down-regulation of p27 protein involves translational inhibition mechanisms, possibly dependent on the corresponding increased accumulation of miR-221/222. Note that expression of p27 is probably a combination of transcriptional and post-transcriptional mechanisms, in keeping with previous reports showing that inhibition of p27 accumulation in Src-transformed fibroblasts is due to reduced transcription and to protein destabilization [26], [27].

**Figure 4:**
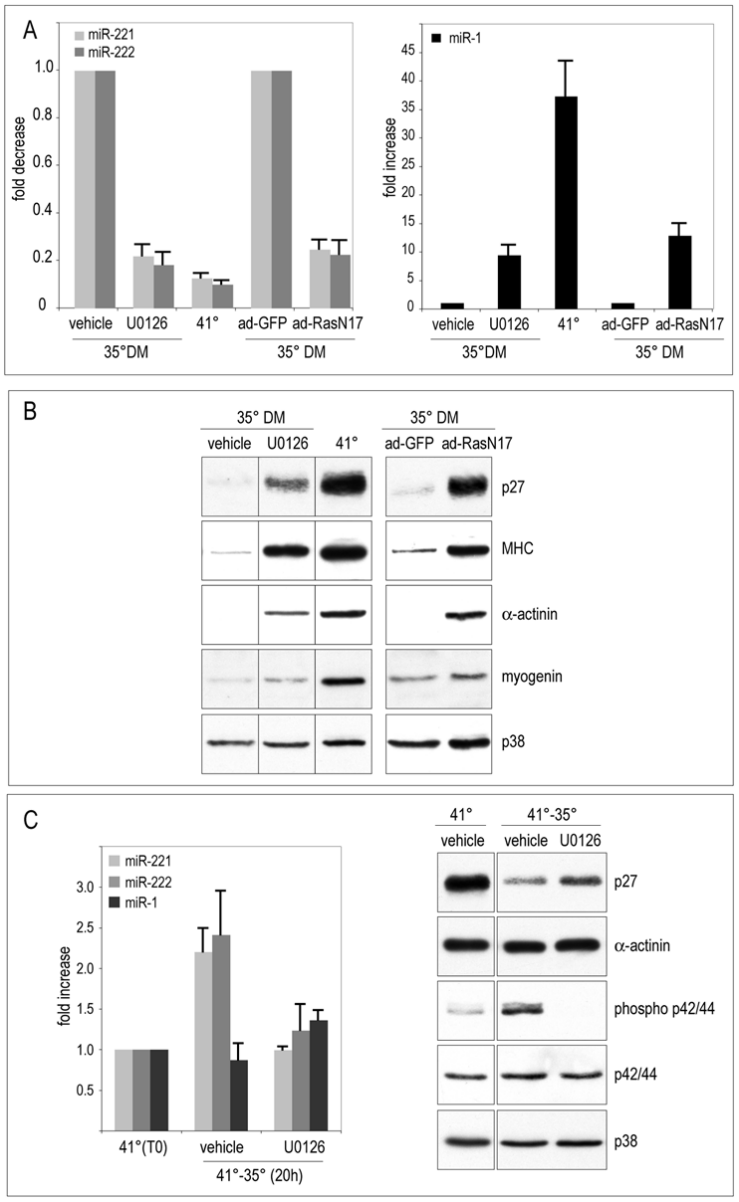
Expression of miR-221 and miR-222 is under the control of the Ras-MAPK pathway. (A, B) Proliferating QMb-ts were treated in DM at 35°C for two days with DMSO (vehicle) or with the MEK inhibitor U0126 (U0126), or infected with adenoviruses expressing GFP (ad-GFP) or a dominant interfering Ras mutant (ad-RasN17). QMb-ts in DM at 41°C are shown for comparison. After 2 days, cells were analysed for (A) expression of miR-221, miR-222 and miR-1 by real time RT-PCR and (B) accumulation of p27 and muscle-specific proteins by western blot. (C) QMb-ts myotubes kept at 41° for 30 hours (41° (T0)) were treated with DMSO (vehicle) or U0126 (U0126) and shifted to 35° for 20 hours. Fold modulation of miRNA expression shown in (A) and (C), normalized for miR-16 expression, is expressed relative to cell extracts at 35°C (A) and myotubes differentiated for 30 hours at 41°C (C), taken as 1.0. Error bars represent standard error and the observed differences are statistically significant (p<0.05; n≥3).

We have recently reported that activation of the Ras-MAPK pathway by Src in quail myogenic cells is largely responsible for inhibition of differentiation both in myoblasts and in post-mitotic myocytes [16]. In this context, we investigated whether this pathway was also involved in the regulation of miR-221/222 expression. Therefore, QMb-ts were treated at 35°C with the MEK inhibitor U0126, or infected with adenoviruses expressing RasN17, a dominant negative Ras mutant, or GFP as control. After 2 days, myoblasts were analyzed for expression of miR-221/222 and miR-1 and for the accumulation of p27 and muscle-specific proteins. Inhibition of the Ras-MAPK pathway by RasN17 or U0126 efficiently inhibited accumulation of miR-221/222 and induced expression of miR-1 (Fig. 4A) concomitantly to that of p27 protein and sarcomeric myosin and α-actinin (Fig. 4B). Note that the levels of miR-221/222 following block of Ras-MAPK pathway are comparable to those observed in myotubes at 41°C, further supporting the central role played by the Ras-MAPK pathway in controlling expression of miR-221/222 downstream of Src. In order to verify whether this pathway was involved in miR-221/222 regulation independently of proliferation, QMb-ts myotubes were subjected to Src activation in the presence of U0126 and analyzed for the expression of miR-221/222 and miR-1. As shown in Fig. 4C, inhibition of the Ras-MAPK pathway in post-mitotic myotubes resulted in expression levels of miR-221/222 comparable to those of myotubes, counteracting the increase observed following Src activation. Interestingly, in contrast to miR-221/222, expression of miR-1 was relatively insensitive to both Src activity (see also Fig. 1) and inhibition of the Ras-MAPK pathway in myotubes (Fig. 4C). Concerning p27, the inhibition of the Ras-MAPK pathway reduced only in part the decrease caused by Src activation in myotubes, (Fig. 4C), probably due to protein destabilization operated by the Src kinase [26]. The efficacy of the treatment with U0126 and of Src activation was monitored by measuring the levels of phosphorylated p42/44 MAPK since no changes in muscle-specific protein accumulation are detectable in myotubes 24 hours after Src activation [18]. These results clearly indicate that the Ras-MAPK pathway regulates expression of miR-221/222 independently of proliferation.

### p27 is a target of miR-221 and miR-222 in avian and mammalian myoblasts

**Figure 5:**
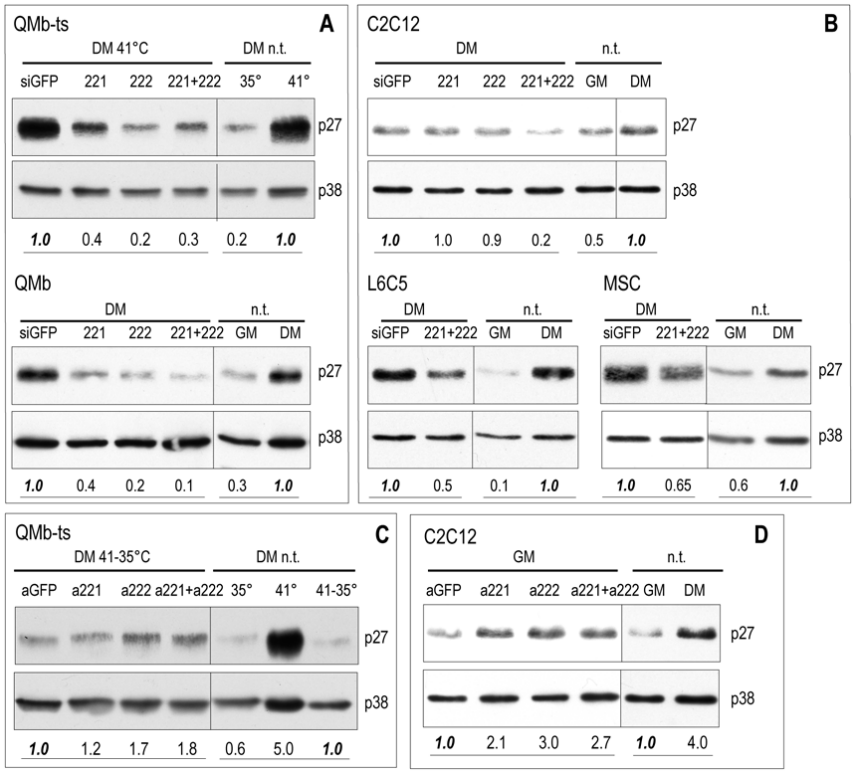
Over-expression and inhibition of miR-221 and miR-222 result in modulation of p27. Western blot analysis of p27 in myotubes derived from (A) QMb-ts and primary QMb and (B) C2C12, L6C5 and MSC myoblasts transfected with control siGFP (siGFP), miR-221 (221), miR-222 (222) or both (221+222) duplex RNAs and allowed to differentiate for two days. p27 expression in (C) QMb-ts myotubes transfected with antisense inhibitors to GFP (aGFP), used as control, miR-221 (a221), miR-222 (a222) or both (a221+a222) and shifted to 35°C for 24 hours and in (D) C2C12 myoblasts transfected with the same duplexes in GM and analysed 24 hours. Untransfected (n.t.) myoblasts in DM at 35°C or GM and (n.t.) myotubes in DM are shown as controls. Numbers represent levels of p27 expressed as fold decrease/increase relative to that measured in controls (siGFP), taken as 1.0. p38 protein levels were used for normalization.

Since the inverse regulation of expression of p27 protein and miR-221/222 was compatible with p27 mRNA being a target of these miRNAs also in myoblasts, we addressed this point by over-expressing or inhibiting miR-221/222 in a variety of avian and mammalian cell contexts. Therefore, QMb-ts, QMb, C2C12, L6C5 and MSC myoblasts were transfected with miR-221, miR-222 or control (siGFP) duplex RNAs, induced to differentiate in DM for two days and analyzed for the expression of p27 protein. Accumulation of p27 was strongly inhibited by both miRNAs upon differentiation of normal and Src-transformed quail myoblasts (Fig. 5A) and significantly reduced in mammalian myocytes (Fig. 5B). Accumulation of p27 mRNA in the same cells was reduced 2–3 fold (not shown), in keeping with the recent finding that miR-dependent translational repression is often accompanied by mRNA destabilization [28], [29]. We then analyzed the consequence of inhibiting endogenous miR-221/222 on accumulation of p27. In order to best evaluate the effects of miR-221/222 inhibition, antisense miRNA inhibitors were transfected in QMb-ts myotubes shifted to 35°C, and in proliferating C2C12 myoblasts, cell contexts where the levels of p27 protein, but not of p27 mRNA, are low (see Fig. 3 and [4]). In both cell types, inhibition of miR-221/222 resulted in a partial recovery of p27 accumulation (Fig. 5C,D), suggesting that p27 mRNA is regulated by these miRNAs in myogenic cells.

**Figure 6:**
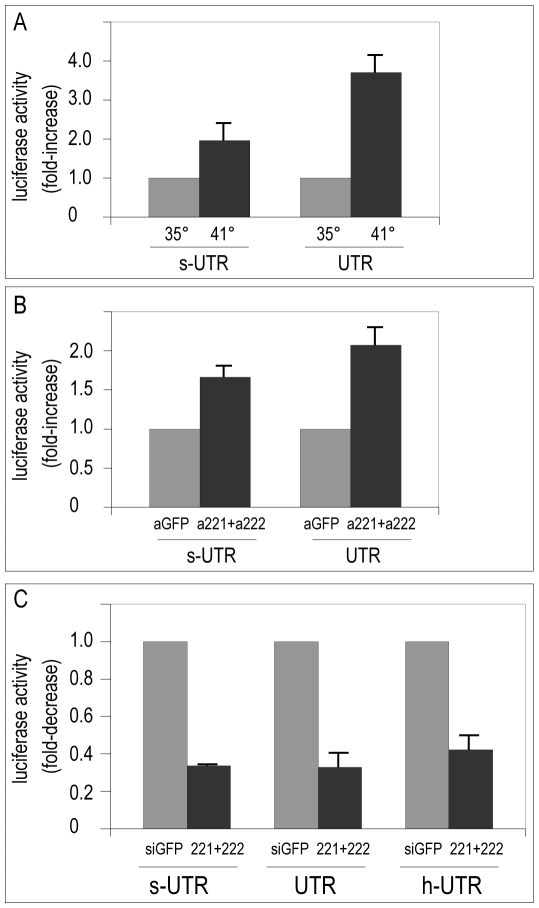
The 3′UTR of quail p27 mRNA is a direct target of miR-221 and miR-222. Firefly luciferase activity was measured in QMb-ts after transfection of pGL3 reporter vectors carrying the entire (UTR) or a portion (s-UTR) of the quail p27-3′UTR containing the target sites for miR-221 and miR-222. (A) pGL3 reporter vectors were transfected and luciferase activities were measured in myoblasts (35°) and myotubes (41°) cultivated in DM; (B) pGL3 reporter vectors were cotransfected with antisense inhibitors to GFP (aGFP) and miR-221 and miR-222 (a221+a222) in myoblasts at 35°C in DM; (C) pGL3 reporter vectors were cotransfected with control (GFP) or miR-221 and miR-222 (221+222) duplexes in myoblasts at 35°C. In (C) also a human p27-3′UTR (h-UTR) is shown for comparison. The transfection efficiency was accounted for by cotransfection with the renilla luciferase reporter pRL. Activities of UTR, s-UTR and h-UTR were normalized on activity of pGL3 empty vector transfected in parallel plates, and expressed relative to activity at 35° (A), to activity of control aGFP (B) and control GFP (C), taken as 1.0. Error bars represent standard error and the observed differences are all statistically significant (p<0.05; n≥3).

To definitively validate quail p27 mRNA as a target of miR-221/222, the full-length p27-3′UTR of quail mRNA and a shorter portion containing two identified target sites for these miRNAs were cloned downstream to the luciferase coding region of the pGL3 reporter vector. Control pGL3 reporter vector or pGL3 containing the short portion (s-UTR) or the full length (UTR) p27-3′UTR were transfected in QMb-ts myoblasts and luciferase activity was measured both in myoblasts and in myotubes (Fig. 6A). A significant decrease in luciferase activity of both p27-3′UTR constructs was detected in myoblasts compared to myotubes, suggesting that endogenous miR-221/222 are competent for inhibition of p27 mRNA translation in these cells. Co-transfection of myoblasts with the p27-3′UTR reporters along with miR-221/222 antisense inhibitors (Fig. 6B) or with miR-221/222 duplexes (Fig. 6C) further demonstrated that quail p27-3′UTR mRNA is a direct target of miR-221/222 and that inhibition of luciferase accumulation by miR-221/222 is efficiently counteracted by their specific antisense inhibitors. A pGL3 reporter containing the human p27-3′UTR [24] was also co-transfected in myoblasts with miR-221/222 duplexes for comparison and found to be regulated also in avian cells (Fig. 6C). In conclusion, we show that in myogenic cells miR-221 and miR-222 play a critical role in finely regulating p27 protein dosage.

### Ectopic expression of miR-221/222 leads to inhibition of muscle-specific gene expression in myocytes and alters maturation of myotubes

**Figure 7:**
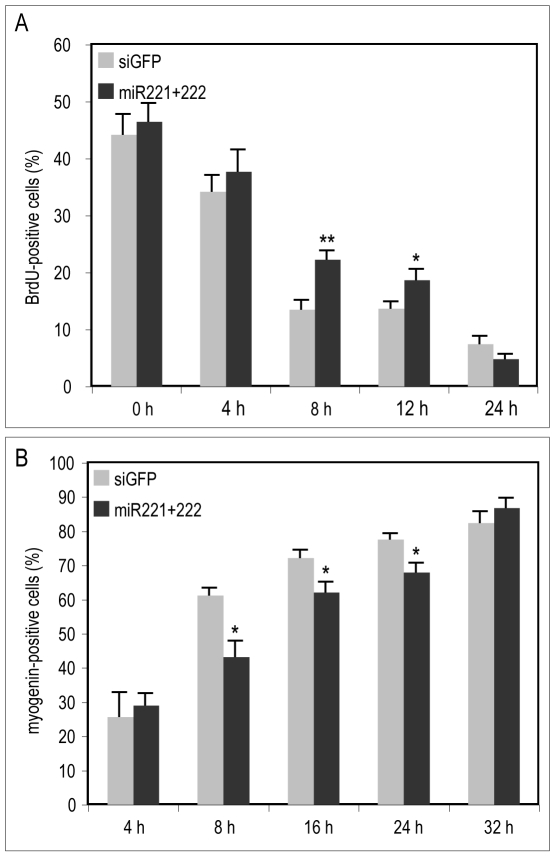
Ectopic expression of miR-221 and miR-222 delays exit from the cell cycle and inhibits myogenin accumulation. QMb-ts were transfected with control (siGFP) or miR-221 and miR-222 duplexes (miR221+222) in GM and transferred to 41°C in DM 24 hours later. At different time points after temperature shift cells were pulse-labeled (for 2 hours) with BrdU, fixed and stained for immunofluorescence with antibodies to (A) BrdU and (B) myogenin. The percentage of positive cells relative to total cells is shown in the histograms. Error bars represent standard error; (*) = p<0.05; (**) = p<0.01; n≥3.

In order to investigate the functional role of miR-221 and miR-222 in progression to terminal differentiation, miR-221/222 were ectopically expressed in proliferating myoblasts and the rate of exit from the cell cycle and accumulation of the early myogenic transcription factor myogenin were measured. QMb-ts myoblasts transfected with miR-221, miR-222 or both and control duplex RNAs at 35°C in GM, were induced to differentiate at 41°C in DM for various lengths of time. At each time point, cells were labeled with BrdU for 2 hours, fixed and processed for immunofluorescence with antibodies to BrdU and myogenin and scored for positive cells. As shown in Fig. 7A and 7B, ectopic expression of miR-221/222 induced a slight, but consistent delay in the exit from the cell cycle up to 12 hours after induction of differentiation and a decrease in the accumulation of myogenin, most evident between 8 and 24 hours.

**Figure 8:**
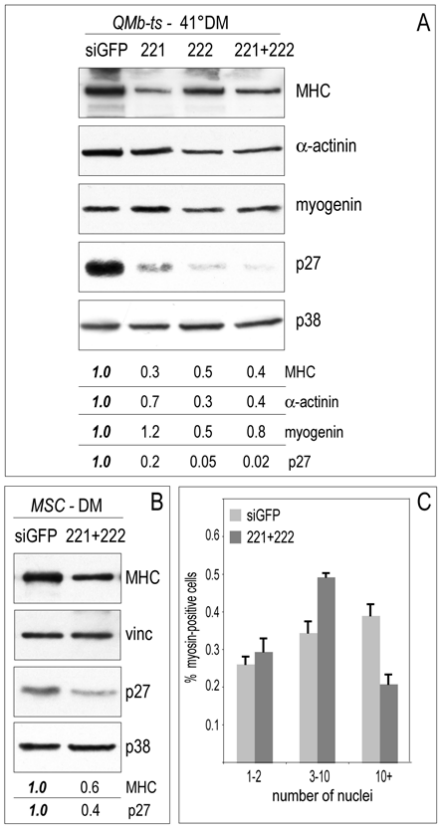
miR-221 and miR-222 reduce muscle-specific protein accumulation and affect cell fusion. (A) QMb-ts transfected at 35°C with control siGFP, miR-221 (221), miR-222 (222) and both (221+222) duplex RNAs were shifted to 41°C in DM and allowed to differentiate for two days. Cell extracts were analyzed by immunoblotting with antibodies specific for the indicated proteins. (B, C) MSC myocytes kept in DM for 24 hours were transfected with control siGFP, miR-221 and miR-222 (221+222), and, after 2 days, were either analyzed by Western blot with antibodies to the proteins indicated in (B) or processed for immunofluorescence and scored for degree of cell fusion (C). Protein accumulation levels expressed as fold decrease/increase relative to those measured in control siGFP, taken as 1.0, are shown in (A, B). p38 and vinculin protein levels were used for normalization. (C) Histogram displaying the size distribution of MSC myotubes, labeled with antibody to myosin heavy chain and with the fluorescent dye Hoechst 33258, expressed as function of the number of nuclei per myotube. Error bars represent standard error.

**Figure 9:**
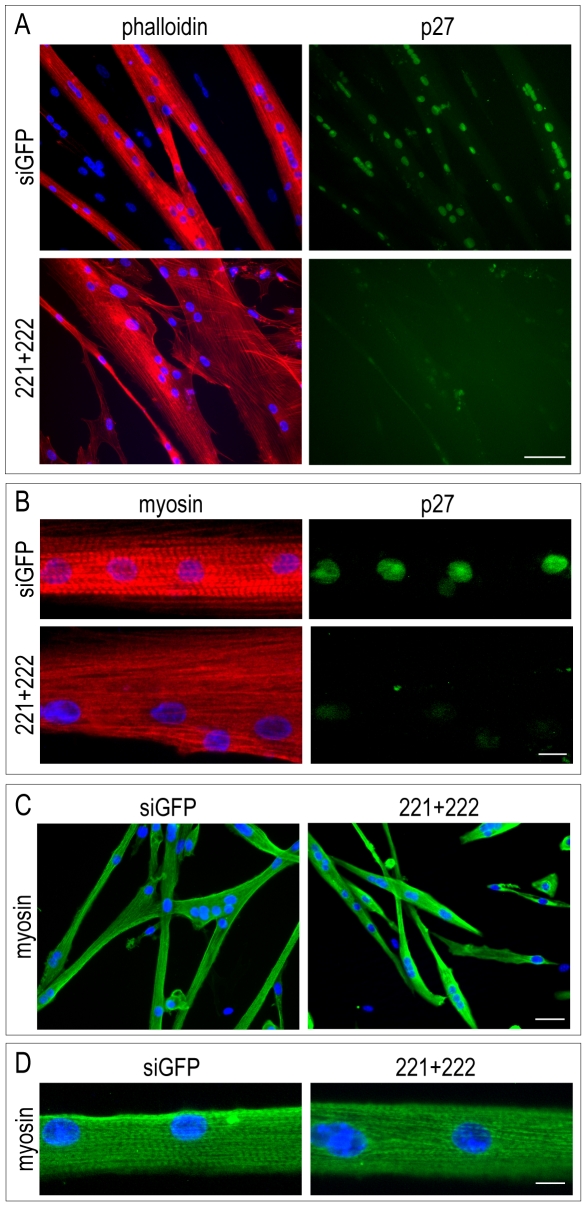
miR-221 and miR-222 induce alterations of myotube morphology and of myofibrillar organization. (A) QMb-ts myoblasts were transfected with control duplexes (siGFP) or a mixture of miR-221 and miR-222 duplexes (221+222), induced to differentiate in DM at 41°C and, two days later, subjected to double immunofluorescence with actin-staining phalloidin and with antibodies specific for p27, as indicated. (B) QMb-ts myotubes, differentiated in DM at 41°C for 24 hours, were transfected with control duplexes (siGFP) or a mixture of miR-221 and miR-222 duplexes (221+222), maintained in DM at 41°C for further 30 hours and subjected to double immunofluorescence with antibodies specific for skeletal myosin and p27, as indicated. (C–D) MSC myocytes, differentiated in DM for 24 hours, were transfected with control duplexes (siGFP) or a mixture of miR-221 and miR-222 duplexes (221+222), maintained in DM for additional 2 days and subjected to immunofluorescence with antibodies specific for skeletal myosin. (A–D) Nuclei were counterstained with Hoechst dye. Note that in (B) and (D) high magnification micrographs are shown to allow visualization of sarcomeres. Scale bars: 40 µm (A, C); 10 µm (B–D).

In order to establish whether the delay in myogenin accumulation influenced the accumulation of sarcomeric proteins, QMb-ts myoblasts overexpressing miR-221/222 were allowed to differentiate for two days and cell extracts were analyzed by Western blot. As shown in Fig. 8A, a significant reduction in the accumulation of muscle-specific myosin heavy chain and α-actinin was observed concomitant to p27 protein reduction. This reduced accumulation of myofibrillar proteins was accompanied by an altered cellular morphology, characterized by widening and flattening of myotubes and loss of alignment and clustering of nuclei (Fig. 9A). To investigate whether the unscheduled expression of miR-221/222 in post-mitotic myocytes could lead to alterations in myotube maturation, QMb-ts and MSC myocytes were transfected with miR221/222 and, three days later, analyzed for the expression of p27, muscle-specific myosin and cell morphology. As observed in QMb-ts myoblasts undergoing differentiation, ectopic expression of miR-221/222 in post-mitotic myocytes induced a reduction of p27 (Fig. 8B and Fig. 9B) and myosin accumulation (Fig. 8B and not shown), accompanied, in MSC, by diminished cell fusion (Fig. 9C), as emphasized by the high percentage of myotubes with a small number of nuclei, compared to control (Fig. 8C).

A specific trait of the terminally differentiated muscle phenotype is the assembly of a highly ordered array of myofibrils. Therefore, organization of myofibrils was analyzed after transfection of miR-221/222 in 1-day-old QMb-ts and MSC myocytes undergoing maturation. As shown in Fig. 9B, QMb-ts myotubes expressing miR-221/222, recognizable by the reduced levels of p27 protein in nuclei, displayed misalignment of myofibrils and reduced sarcomere assembly, highlighted by the poor cross-striation pattern evidenced by myosin staining, as compared to controls transfected with siGFP. A similar phenotype was observed in MSC myotubes expressing miR-221/222 (Fig. 9D). Note that monoclonal antibody (mAb) to p27 does not recognize the antigen in MSC by immunofluorescence. Levels of p27, therefore, were routinely measured in parallel cultures by western blot analysis, as in Fig. 8B. Altogether these findings strengthen the notion that the expression of miR-221 and miR-222 needs to be tightly regulated during differentiation and suggest the existence of a role of these miRNAs both in progression of myogenic differentiation and in achievement of the differentiated phenotype.

## Discussion

Beside the well-defined transcriptional level of regulation, the discovery of miRNAs adds a new layer of post-transcriptional mechanisms that appear to fine tune protein dosages of key regulators during muscle differentiation. Importantly, the emerging crucial role of miRNAs in animal development suggests that these RNAs might also be involved in muscle diseases and, indeed, recent studies have shown that deregulation of miRNAs is linked to skeletal and cardiac hypertrophy and muscular dystrophy [30]–[32]. By comparing miRNA expression profiling of proliferating quail embryo myoblasts and terminally differentiated myotubes, we identified a number of miRNAs strongly modulated upon differentiation that had not been previously described in other myogenic cell contexts. Two of them in particular, miR-221 and miR-222, were down-regulated during differentiation in all myogenic cells examined. Expression of miR-221 and miR-222 was restored in post-mitotic avian myotubes subjected to activation of thermolabile Src kinase, a finding that correlates with the consistently elevated levels of miR-222 expression observed in human muscle disorders [31] and in muscles of 3- and 18-months-old *mdx* dystrophic mice (B. C., L. C. and G. F., unpublished observations). Together, these findings strongly suggest that miR-222 plays a role in muscle cell damage. miR-221 and miR-222 are both up-regulated in several tumor-derived cell lines and in cancer patients and recent studies suggest that miR-221 and miR-222 promote cell cycle progression of tumor cells [33]–[35]. Interestingly, we found that expression of miR-221/222 can be induced in a post-mitotic context, thus in a proliferation-independent fashion.

miR-221/222 have been shown to control protein levels of the cell-cycle inhibitors p27 and p57 [22]–[24], [36], [37]. Here we report that also in myogenic cells the coordinated expression of miR-221 and miR-222 inversely correlates with the expression of p27 and demonstrate that p27 is a direct target of both miRNAs in myoblasts. In contrast, no down-modulation of p57 was observed in myotubes from mouse myoblasts over-expressing miR-221/222; moreover, the avian p57 mRNA homologue does not appear to harbor putative target sequences for miR-221 and miR-222 (B. C., L. C. and G. F., unpublished observations). Over-expression of miR-221/222 in myoblasts undergoing differentiation, while hampering p27 accumulation and inducing a transient delay in the exit from the cell cycle, was not sufficient to maintain myoblast proliferation. Our findings are in keeping with the observation that in tumor cells reduced expression of p27 is not sufficient to sustain active cell proliferation in the absence of growth factors [37].

Beyond contributing to modulation of cell proliferation, novel regulatory functions of miR-221 and miR-222 are described in progression and achievement of full myogenic differentiation. Indeed, forced expression of miR-221/222 both in myoblasts induced to differentiate and in post-mitotic myocytes caused a diminished accumulation of muscle specific proteins and modification of cellular morphology. Moreover, both avian and mammalian myotubes displayed defects in sarcomere assembly, a specific trait of the fully differentiated phenotype. Having established that p27 is a target of miR-221 and miR-222 in myoblasts and is down-modulated by miR-221/222 unscheduled expression in post-mitotic myocytes, the question arises of whether inhibition of p27 expression accounts for the observed defects of myogenic differentiation. It was previously reported that abrogation of p27 in C2C12 myoblasts reduces fusion and expression of muscle myosin [4], although overall morphology and assembly of sarcomeric structures were not analyzed. It is noteworthy that, beyond control of cell cycle, p27 may regulate several other functions both in myoblasts and in other cell types, such as cadherin-mediated cell-cell adhesion [4], tumor progression, apoptosis and cytoskeletal dynamics [38]. Interestingly, a negative feedback loop has been described to occur between p27 and RhoA, a pivotal regulator of the actin cytoskeleton [38], and over-expression in myotubes of RhoA leads to alterations strikingly similar to those induced by ectopic expression of miR-221 and miR-222 (L.C. and G.F., unpublished observations). Therefore, it is possible that the alterations of morphology and sarcomere assembly that we observe in myotubes expressing ectopic miR-221 and miR-222 are mediated by down-modulation of cell cycle-independent functions of p27 and/or by inhibition of other targets.

Being the miRNA world a relatively recent field of investigation, very little is known about the pathways regulating the expression and function of these RNAs. It was recently reported that signalling through the ERK MAPK delayed expression of muscle-specific miRNAs in C2C12 myoblasts undergoing differentiation [11]. Here we describe for the first time that in QMb-ts myoblasts induced to differentiate and in post-mitotic myotubes the expression of miR-221 and miR-222 is under the positive control of the Ras-MAPK signalling pathway, independently of proliferation. This well correlates with the finding that inhibition of the Ras-MAPK pathway in QMb-ts myoblasts induced the expression of p27 and muscle-specific proteins and the ability to fuse and assemble contractile structures [16]. We confirm that the Ras-MAPK pathway negatively regulates expression of miR-1 in myoblasts, but we show that this miRNA is relatively insensitive to this pathway in myotubes subjected to Src activation. Src and the Ras-MAPK pathway have been implicated in repression of MyoD and myogenin transcriptional activity in myoblasts [17], [39]–[41] and, recently, MRFs have been shown to regulate transcription of muscle-restricted miR-1, miR-133 and miR-206 [10], [11], likely by direct binding to their presumptive promoters [10]. In turn, muscle-specific miRNAs control accumulation of inhibitors of muscle gene expression, thereby establishing positive feedback loops resulting in enhanced differentiation [7]. Therefore, the modulation of miRNA expression observed in QMb-ts may represent a mechanism linking cytoplasmic intracellular signaling initiated by Src through MAPK-pathway with nuclear events leading to transcriptional inhibition of muscle gene expression [39]. It is becoming clear that miRNAs are important elements of the differentiation program of skeletal muscle cells. How the interactions between upstream regulators of miRNAs and the targets of miRNA activity lead to muscle differentiation and to its maintenance will require further investigation.

## Materials and Methods

### Ethic Statement

All animals were handled in strict accordance with good animal practice as defined by the D.L 116/1992 of the Ministero della Salute (Italy), and all animal work was approved by the Ministero della Salute, Dipartimento Alimenti, Nutrizione e Sanità Pubblica Veterinaria- Ufficio X°.

### Materials and antibodies

Highly purified Triton X-100 and NP-40 were from Roche. FITC and TRITC-conjugated phalloidin and mAb to vinculin (VIN-11-5) were from Sigma. mAb to BrdU (BU-1) was from GE Healthcare. mAbs to p27 and to Ras (cl.18) were from Transduction Laboratories. mAb to skeletal α-actinin (9A2B8) and myosin heavy chain (MF20) were obtained from D. Fischman. Rabbit serum to chicken myogenin was provided by B. Paterson. A rabbit serum to chicken skeletal muscle myosin was developed in house [39]. Rabbit polyclonal antibodies to p38 were from Santa Cruz Biotechnology and to phospho- and total p42/44 MAPK were from Cell Signaling. FITC- and TRITC-conjugated goat anti-rabbit and anti-mouse antibodies were from Jackson ImmunoResearch Laboratories. Horseradish peroxidase-conjugated goat anti-mouse and anti-rabbit antibodies were from Bio-Rad.

### Plasmid construction, RNA duplexes and 2′-O-Methyl RNA oligonucleotides

To construct the p27-3′UTR luciferase reporter plasmids (UTR and s-UTR), the full length and a portion of the 3′UTR of quail p27 mRNA were amplified by RT-PCR from total RNA of quail myotubes using the following primers obtained from the 3′UTR sequence of chicken p27 mRNA (Genbank Accession Number NM_204256) and containing the XbaI restriction site:

Forward: 5′-TATTCTAACTCCCTAAGGCGGAGGACT-3′

Reverse 1: 5′-TATTCTAGAACAGGGGACCCACTTAAAGG-3′

Reverse 2: 5′-TATTCTAGAACATACAGGTACACAGGCAATG-3′

The 3′UTR PCR products were sequenced and the nucleotide sequence deposited in the Genbank database (Genbank Accession Number FJ378653). The fragments were ligated into the XbaI site downstream to the firefly luciferase coding sequence of the pGL3-Promoter vector (Promega).

The following sequences of the miRNA duplexes were designed according to [42]:

miR-221 sense: 5′-AGCUACAUUGUCUGCUGGGUUUC-3′

miR-221 antisense: 5′-AACCCAGCAGACAAUGUAGUUUU-3′

miR-222 sense: 5′-AGCUACAUCUGGCUACUGGGUCUC-3′

miR-222 antisense: 5′-GACCCAGUAGCCAGAUGUAGCUUU-3′

The miRNA duplexes are siRNA-like miRNA mimics that have been shown to function on target mRNAs as well as endogenous miRNAs [43]. A Green Fluorescent protein siRNA duplex (Eurofins) was used as control (siGFP).

The sequences of the 2′-*O*-Me antisense RNA oligonucleotides were the following:

2′-*O*-Me anti miR-221: 5′-GAAACCCAGCAGACAAUGUAGCU-3′

2′-*O*-Me anti miR-222: 5′-GAGACCCAGUAGCCAGAUGUAGCU-3′

2′-*O*-Me anti GFP: 5′-UCUUCGGCAAGCUGACCCUGAAGUUACCUU-3′

### Cell cultures and viral infection

Primary cultures of quail myoblasts (QMb) were prepared as described previously [19] and maintained proliferating in DMEM supplemented with 10% FCS, 10% tryptose phosphate broth and 3% quail embryo extract (GM) at 37°C. Polyclonal populations of quail myoblasts transformed by the temperature-sensitive mutant of the Rous Sarcoma Virus LA29 (QMb-ts) were established as described [19] and propagated at 35°C in GM devoid of quail embryo extract. Differentiation was induced by plating the cells on collagen-coated dishes in GM and, the following day, by substituting GM with DMEM supplemented with 2% FCS (DM) and incubating the cell at 41°C. C2C12 [44] and L6C5 myoblasts [45] (provided by M. Grossi) were maintained proliferating at 37°C in DMEM supplemented with 15% FCS (GM). Differentiation of C2C12 myoblasts was induced by incubating the cultures in DMEM with 2% horse serum (DM) and of L6C5 myoblasts by incubating the cells in DMEM with 1% FCS and 1 mg/ml of insulin. Primary mouse satellite cells (MSC) [46] (provided by D. Pajalunga) were grown in Ham's medium supplemented with 20% FCS, 3% chicken embryo extract and 2.5 ng/ml bFGF. Differentiation was induced by incubating the cultures in DMEM with 10% FCS.

High-titer stocks of recombinant adenoviruses expressing GFP (provided by M. Crescenzi) or RasN17 (provided by L. Parada) were used to infect QMb-ts cells. Virus expression was scored by immunofluorescence and only cultures showing a percentage of infection over 70% were further processed. For treatment with MEK inhibitor U0126 (Promega), myoblasts were kept for 2 days at 35°C in DM containing 12.5 µM U0126 or vehicle (DMSO) and medium was renewed every day; myotubes differentiated for 30 hours at 41°C were shifted to 35°C in DM containing 12.5 µM U0126 or vehicle (DMSO) for 20 hours.

### miRNA quantification

Total RNA was extracted using TRIzol (Invitrogen). RNA enriched for small RNAs was obtained using the PureLink miRNA Isolation Kit (Invitrogen), according to the manufacturer's instructions. miRNA levels were analyzed using the TaqMan Real Time PCR method (1 ng/assay), and quantified with ABI Prism 7000 SDS (Applied Biosystems). Primers for miRNAs and the reagents for reverse transcription and PCR reactions were all obtained from Applied Biosystems. Relative expression was calculated using the comparative Ct method (2^−ΔΔCt^) [47]. Different samples were normalized to miR-16 expression. Conditions for miRNA profiling were previously described [48]. Briefly, to detect mature miRNA species, reverse transcription and amplification were performed with specific primers and TaqMan probes. 250 miRNAs were assayed in a 96-well format and samples were also normalized to the median Ct value. Heat maps were generated using Genesis software 1.7.2 version (Graz University of Technology).

### RNA expression analysis

Total RNA (30 µg) and RNA enriched for small RNAs (2 µg) was separated through a 12% denaturing urea-polyacrilamide gel and transferred to a GeneScreen Plus nylon membrane. miR-221, miR-222, miR-133 probes (DNA oligonucleotides complementary to the microRNA sequences) and U6 probes were end labeled with 50 µCi of [γ-^32^P]dATP. Membranes were hybridized in 5X SSPE, 10% Dextran Sulphate, 5X Denhartd solution, 1% SDS and 50% formamide at 37°C for 18 hours and washed in 2X SSPE and 0.2% SDS twice at room temperature and once at 37°C. Bands were visualized on a PhosphorImager (Molecular Dynamics) and quantified using ImageQuant 5.1 software (Molecular Dynamics). For quantitative RT-PCR analysis of p27 mRNA, total RNA was extracted using the TRIzol reagent and retro-transcribed with the Reverse Transcription System (Promega) using oligo (dT). Primers for quail p27 mRNA analysis were: (Forward) 5′-AGCAAACACCCAAGAAATCGA-3′ and (Reverse) 5′-CTCCGCCTTAGGGAGTTTACG-3′; primers for quail GAPDH mRNA analysis were: (Forward) 5′-GAGGGTAGTGAAGGCTGCTG-3′ and (Reverse) 5′-CCACAACACGGTTGCTGTAT-3′. Analyses were carried out using Power SYBR Green PCR master mix (Applied Biosystems) and ABI Prism 7500. Results were normalized with respect to GAPDH expression. Relative expression was calculated using the comparative Ct method (2^−ΔΔCt^) [47].

### Transient transfections and luciferase assays

QMb, QMb-ts, C2C12 and L6C5 myoblasts were transfected with 100–200 nM duplex RNAs or 50–100 nM antisense RNA oligos using the lipofectamine reagent (Invitrogen) in serum-free Optimem (Gibco-BRL). QMb and QMb-ts myoblasts were transfected at 35°C for 4 hours, fed with fresh GM and, 24 hours later, transferred to DM at 41°C for different times. QMb-ts myotubes, kept at 41°C in DM for at least 24 hours, were transfected for 4 hours and maintained at 41°C in DM for further 24 hours. C2C12 and L6C5 myoblasts were transfected at 37°C in GM and, after 4 hours, medium was replaced with fresh GM and maintained for a minimum of 24 hours, or shifted to DM for at least 2 days. MSC were transfected with 50 nM duplex RNAs using the HiPerfect reagent (Qiagen). Transfection was performed either when medium was replaced with DM or after 24 hours in DM. Transfection efficiency of miRNA duplex oligonucleotides was monitored in each experiment by real time PCR quantification and miR-221/222 levels were found to be at least 400-fold higher than the endogenous miRNA levels.

For the expression of luciferase reporter constructs, 50 ng of pGL3 (Promega) and pGL3-derived plasmids were co-transfected with 50 ng of pRL-TK (Promega) per 10^5^ cells to normalize for transfection efficiency using the lipofectamine reagent in serum-free Optimem. In some experiments, a pGL3 construct containing the human p27mRNA-3′UTR (h-UTR, obtained by A. Fusco, [24]) was also used. When required, 50–100 nM of duplex or antisense RNAs were co-transfected with plasmid DNA. QMb-ts myoblasts were transfected at 35°C, transferred in DM, and then either kept at 35°C or shifted to 41°C to induce differentiation. 24–48 hours later, cells were lysed and luciferase expression was measured with the Dual Luciferase Assay kit (Promega) using a luminometer (Lumat LB9507, Berthold).

### Bromo-deoxyuridine labeling and immunofluorescence analysis

For bromo-deoxyuridine (BrdU) incorporation studies, QMb-ts transfected at 35°C were shifted to 41°C in DM and labeled with 20 µM BrdU for 2 hours at various times following temperature shift, as required. Following BrdU incorporation, cells were fixed and processed for immunofluorescence. Cultures to be analyzed by immunofluorescence were routinely fixed with 4% paraformaldehyde, permeabilized with 0.25% TritonX-100 in Phosphate Buffered Saline and processed as previously described [39]. For labeling with rabbit polyclonal antibodies to myosin, following fixation with paraformaldehyde, cells were permeabilized with ice-cold 100% methanol and washed thoroughly before incubation with antibodies. For labeling with mAb to myosin heavy chain (MF20), cultures were fixed with a mixture of 3.5% formaldehyde, 70% ethanol and 5% acidic acid and washed thoroughly before incubation with antibody. The samples were examined with an Olympus microscope. Images were recorded on a CCD camera and processed using a DeltaSystem and Adobe Photoshop software. Cell scoring was carried out using the public domain software ImageJ.

### Whole-cell extracts and Western blot analysis

Cells were lysed in RIPA buffer (140 mM NaCl, 3 mM MgCl2, 1 mM EDTA, 1 mM orthovanadate, 15 mM Hepes, pH 7.2, also containing 0.5% Nadeoxycholate, 1% NP-40, 0.1% SDS) supplemented with a cocktail of protease inhibitors. Western blots were carried out using horseradish peroxidase-conjugated goat anti-rabbit and anti-mouse antibodies and revealed with a chemiluminescence detection system by Pierce. Quantitation of the bands was carried out by scanning films using the CanoScan D2400U by Canon and evaluating band intensity using ImageQuant software.

### Statistical Analysis

Variables were analyzed by both Student's t test and one way ANOVA and a probability value of p ≤0.05 was deemed statistically significant. Values are expressed as average ± standard error (s.e.).

## Supporting Information

Table S1 MiRNA Expression Profiling in Quail Myoblasts Expressing ts-Src Kinase
(0.23 MB XLS)
